# Supplementary material for: Scalable multifunctional MOFs-textiles via diazonium chemistry
Source: Nat Commun. 2024 Jun 21;15:5297. doi: 10.1038/s41467-024-49636-9 (PMC11192900; doi:10.1038/s41467-024-49636-9)
Supplement: Supplementary file 1 — Supplementary Information [file 41467_2024_49636_MOESM1_ESM.pdf]

Supplementary Information

Scalable multifunctional MOFs-textiles via diazonium chemistry

Wulong Li[1,2], Zhen Yu[3], Yaoxin Zhang[4], Cun Lv[5], Xiaoxiang He[5], Shuai Wang[1], Zhixun Wang[1], Bing He[1], Shixing Yuan[1], Jiwu Xin[1], Yanting Liu[1], Tianzhu Zhou[1], Zhanxiong Li[5,6*], Swee Ching Tan[2*], and Lei Wei[1*]

[1]School of Electrical and Electronic Engineering, Nanyang Technological University, Singapore, 639798, Singapore.

[2]Department of Materials Science and Engineering, National University of Singapore, Singapore 117575, Singapore.

[3]School of Environmental Science and Engineering, Tianjin Key Lab of Biomass/Wastes Utilization, Tianjin University, Tianjin, 300072, China.

[4]China-UK Low Carbon College, Shanghai Jiao Tong University, Shanghai, 201306, China.

[5]College of Textile and Clothing Engineering, Soochow University, Suzhou, 215021, China.

[6]National Engineering Laboratory for Modern Silk, Soochow University, Suzhou, 215021, China.

*Corresponding authors.

E-mail: lizhanxiong@suda.edu.cn, msetansc@nus.edu.sg, wei.lei@ntu.edu.sg

This supplementary information file includes:

Supplementary Notes 1–8

Supplementary Figures 1–37

Supplementary References 1–27

Supplementary Notes

Supplementary Note 1: Comparison of the ZIF-67-CT with pristine cotton textiles, conventionally modified cotton textiles, and other ZIF-67 coating textiles.

In Fig. 1b, we compare the ZIF-67-CT with pristine cotton textiles, conventionally modified cotton textiles, and other ZIF-67 coating textiles in terms of scalability, mechanical stability, porosity, fragrance encapsulation, pollutant degradation, and antibacterial activity:

Scalability

The ZIF-67-CT is fabricated using two steps modification method: first, the carboxymethylation of cotton textile (CT) via diazonium chemistry; second, ZIF-67 in situ growth on the fiber surface of carboxymethylated cotton textile, and washing and drying; the two steps fabricating processes are finished at room temperature (20~30 °C) and highly scalable. However, the preparation of functional textiles by conventionally modified cotton textiles, such as those that require the use of complex chemical reactions and lots of organic solvents[1,2] or other high-cost techniques by requiring high-temperature treatment, special equipment, and high voltage[3-6]. The complexity and/or high cost make these methods difficult to scale up. Additionally, most other ZIF-67 coating textile techniques are limited only in the lab preparation[7,8].

Mechanical stability

For the ZIF-67-CT, the hierarchical structures of the cotton textiles are not significantly damaged during the modified processing due to the low-temperature reactive condition, hence the tensile strength is lower than that of the pristine textiles, but higher than that of conventionally modified cotton textiles, which is modified at high temperature. More importantly, the coating shows high stability and is able to sustain repeated washing in water. The covalent grafted carboxyl polymer chain brushes on the fiber surface create abundant carboxyl group sites, which assist the initial coordination of cobalt ions to the fiber surface and facilitate the subsequent in situ growth of MOF nanoparticles, forming a uniform and dense ZIF-67 MOF coating. The coordination bonding between ZIF-67 and carboxyl chains of cellulose fibers endows this ZIF-67-textile to be highly stable in air and water. For conventionally modified cotton textiles, most coatings are typically incorporated into the fibers with non-covalent bonds using methods such as dip-coating, hot-pressing, and spraying, which causes problems with the coating with poor wash and wear durability for its low adhesivity, non-covalent bond force, and weak bonding ability (for example, van der Waals forces and weak electrostatic interactions)[9,10].

Porosity

The porosity of ZIF-67-CT and other ZIF-67 coating textiles is higher than unmodified cotton textiles,

which is attributed to the mesoporous structure of ZIF-67 MOF coating on the fiber surface. However, the coatings of most conventionally modified cotton textiles only have rough structures or micro-nano particles, which do not have unique porous structures like MOFs with large specific surface area and tunable pore size, so they cannot significantly increase their porosity.

Fragrance encapsulation

One of the essential merits of MOF materials is their large surface area and high porosity, which enables the encapsulation of small active molecules and sustains the release of payloads via enhanced host-guest interactions[11,12]. Therefore, the ZIF-67-CT exhibits high porosity with greatly improved specific surface area, allowing sufficient encapsulation and controlled release of different functional and beneficial molecules, including essential oils, antibacterial agents, and active drugs. However, the unmodified and conventionally modified cotton textiles cannot be used for fragrance encapsulation, and the other ZIF-67 coating textiles have not been investigated and applied in fragrance encapsulation.

Pollutant degradation

Owing to the reaction rate of PMS in contact with the reactants is quite slow, thus, catalysis is necessary in the reaction system. However, the unmodified and conventionally modified cotton textiles cannot activate the PMS to generate any radical species for the pollutant degradation. In recent years, transition metals (Co^{2+}, Fe^{2+}), metal oxides (Co_3O_4 and $CoFe_2O_4$), and cobalt-based metal-organic frameworks (MOFs) have been used to activate PMS to generate $•SO_4^-$ and •OH[13,14]. In particular, the novel cobalt-based MOFs with good structure stability have received extensive attention because of their large specific surface area and excellent catalytic performance in SR-AOPs. In this work, the ZIF-67-CT can be used as a catalyst for activating PMS to generate $•SO_4^-$ and •OH, resulting in the degradation of contaminants such as MB, RhB, and MO dyes, showing excellent degradation ability (discussed in detail in the main texts).

Antibacterial activity

The antibacterial activity of the ZIF-67-CT and other ZIF-67 coating textiles is attributed to the cobalt ions in the MOF, based on the same working mechanisms as that of conventionally modified cotton textile and other ZIF-67 coating textiles with Co species additives. Interestingly, the carvacrol-loaded ZIF-67-CT shows superior antibacterial activity against bacteria with larger inhibition zones of 33.0 mm against *E. coli* and 90.0 mm against *S. aureus*. This result indicates that the loaded fragrance carvacrol could continuously release the encapsulated molecules into the agar plate inoculated bacteria, benefiting from the large specific surface area and high porosity of ZIF-67 (discussed in detail in the main texts).

Supplementary Note 2: The detailed reaction mechanism of the fabrication of ZIF-67-CT.

The mechanism of the grafting of diazonium salts has been extensively reported in the literature [15-17]: it involves a reductive electron transfer to the diazonium salt concerted with the cleavage of dinitrogen[18] leading to the generation of aryl radicals, followed by the binding of these radicals to the surface of the substrate via covalent bonds[19,20]. The final material obtained consists of an organic layer covalently attached to the surface of the substrate, the thickness of which can be varied from a monolayer[21] to a multilayer [22]. In this work, the detailed reaction mechanism of the fabrication of ZIF-67-CT can be divided into the following steps. Starting the 3-aminobenzoic acid was reacted with an equivalent quantity of $NaNO_2$ in 1 mol L^{-1} of HCl to form 3-aminobenzoic acid diazonium salt, which would be reduced to highly active aryl radical and released N_2 through VC reduction at room temperature (Supplementary Fig. 1a). Then, the hydroxyl groups of cellulose could be initiated a homolytic reaction by the generated aryl radicals to form oxygen radicals on the cellulose backbone[20,23] (Supplementary Fig. 1b). Finally, the aryl radical could covalent bound with oxygen radical on the cellulose backbone, while other free radicals could continue to graft on the already attached aryl ring to form multilayer carboxyl polymer chain brushes on the fiber surface (Supplementary Fig. 1c). As shown in Supplementary Fig. 2, the covalent grafted carboxyl polymer chain brushes on the fiber surface could coordinate abundant cobalt ions and form a saturated ion interface layer, which facilitates the subsequent in situ growth of MOF nanoparticles in metal-contenting and ligand-contenting solution, forming a uniform and dense ZIF-67 MOF coating on the fiber surface.

Supplementary Note 3: The color analyses of prepared textiles.

The textile color variables (color depth K/S, color saturation C*, hue angle h*, lightness coefficient L*, and color parameters of a* and b*) before and after modification were measured using a HunterLab UltraScan PRO (HunterLab, USA). The value of the color depth K/S, color saturation C*, hue angle h*, lightness coefficient L*, and color parameters of a* and b* were recorded. The lightness coefficient 'L*' represents brightness and darkness, the 'a*' value represents greenish and redness as the value increases from negative to positive, and 'b*' represents bluish and yellowish. Hue angle (h*) is derived from a* and b* color space, and therefore is a more practical parameter in reflecting textile color[24].

Different samples displayed significant differences in color parameters. As shown in Supplementary Fig. 5b, Hunter L* showed a gradual decrease, a* gradually increased, while Hunter b* and C* increased and then decreased as the textile color changed from white to yellow, and to purple (Supplementary Fig. 5a). The h* value of CT-COOH is lower than pristine cotton textile CT, and the ZIF-67-CT is higher than both CT-COOH and CT. The higher the hue angle, the more purple the textile. Furthermore, the color absorption spectra of textiles are consistent with the visual textile color and color parameters (Fig. 2i), further confirming the successful modification process of textiles.

Supplementary Note 4: Comparison of MOF in situ growth on unmodified and carboxymethylated cotton fiber surfaces.

As shown in Supplementary Fig. 7, only sparse and patchy particles appeared on the unmodified CT fiber surface, and showed a much lighter and uneven purple color during the same period. Owing to the unmodified cotton fibers being devoid of reactive carboxymethyl groups, there is sparse MOF nucleation forming on the unmodified CT fiber surface. Consequently, MOF nucleation seeding likely occurs on random defects of untreated fiber and continues to grow, leading to sparse and irregular ZIF-67 particles.

In stark contrast to unmodified cotton fibers, the surface of carboxymethylated cotton fibers grafted carboxyl polymer brushes which creates abundant COOH sites for subsequent coordination metal ion reaction, followed by in situ growth of MOFs on the surface of carboxymethylated cellulose fibers (CT-COOH). Therefore, CT-COOH textile introduced abundant carboxyl polymer brushes, which could serve as nucleation center layer and are very beneficial to the in situ growth of MOFs, forming a uniform and dense MOF coating on the fiber surface (Fig. 2c).

Supplementary Note 5: The chemical stability of superhydrophobic ZIF-67-CT.

As shown in Supplementary Fig. 16, the ZIF-67-CT would be sunk to the bottom of the acid and alkali solutions (Supplementary Figs. 16a and b), and the colour of ZIF-67-CT was transformed from purple into a faint yellow after soaking for 48 h at 30 °C. On the contrary, the superhydrophobic ZIF-67-CT completely floated on the water in the bottle (Supplementary Figs. 16a and b) due to the superhydrophobicity, and the color of superhydrophobic ZIF-67-CT is not changed even after soaking under the same conditions. The ZIF-67 crystal structure of ZIF-67-CT was lost after soaking for 48 h (Supplementary Figs. 16b and d), whereas the characteristic peaks of superhydrophobic ZIF-67-CT are well maintained. The time-dependent chemical stability test data results demonstrate that the superhydrophobic modification of ZIF-67-CT shows good acid and alkali resistance stability. The enhancement of chemical stability of superhydrophobic ZIF-67-CT should be due to the formed stable hydrophobic 1H,1H,2H,2H-perfluorooctyltriethoxysilane film on the MOF coating surface, which effectively prevents the chemical solutions contact the MOF structures. Therefore, the possibility of attack from the chemical solutions can be significantly reduced, further improving the superhydrophobic ZIF-67-CT’s good acid and alkali resistance stability.

Supplementary Note 6: The oil-water separation ability.

We tested the oil-water separation ability of superhydrophobic ZIF-67-CT with different oil-water mixtures. Supplementary Fig. 19 shows the separation process of oil-water mixtures such as petroleum ether-water and carbon tetrachloride-water. The oil phase of petroleum ether and carbon tetrachloride (dyed red) could permeate through the superhydrophobic ZIF-67-CT textile filter, but the water (dyed blue) couldn't pass it due to its superhydrophobic and superoleophilic properties. The prepared sample could complete the separation process of the heavy carbon tetrachloride-water mixture by its gravity (Supplementary Fig. 19a). For the light oil petroleum ether-water mixture, we designed an oil absorption bag using the superhydrophobic ZIF-67-CT-wrapped sponge to separate the oil-water mixture. Similarly, the separation process of the petroleum ether-water mixture can be finished by the water pump driven, showing a very high separating rate (Supplementary Fig. 19b). We also tested the recyclable separation ability of superhydrophobic ZIF-67-CT, which is very important for its practical applications. As presented in Supplementary Fig. 19c, the separation efficiency was up to 98.6%, and the separation efficiency still maintained above 97% after 15 cycles separation process, exhibiting excellent recyclability and durability.

Supplementary Note 7: The degradation ability.

As displayed in Supplementary Fig. 22, 50 mg L^{-1} of dye waste water solution mixed with a certain amount of peroxymonosulfate (PMS) is added into a designed simple device to complete the waste water purification separation process, in which the ZIF-67-CT textile as the degradation separation membrane. The degradation effect was measured using a UV-vis absorption spectrophotometer. We first investigated the influence of the PMS concentration on the ZIF-67-CT catalytic degradation. Supplementary Figs. 23, 24, and 25 show photographs, UV-vis absorption spectra, and degradation performance of the MB solution after degradation with six different PMS concentrations (0.0, 0.6, 0.9, 1.2, 1.5, 1.8 mg mL^{-1}), which confirmed that the higher concentration of the PMS, the more colorless of the solution, showing more degradation effect, and the 98.2% degradation efficiency was reached at 1.8 mg mL^{-1} of PMS. In addition, we investigated the dye degradation kinetic of ZIF-67-CT with PMS at room temperature. Supplementary Fig. 26 shows that the ZIF-67-CT could degrade 96.8% of the MB within 8 min, slightly increasing to 99.1% in 10 min. Therefore, we used the 1.8 mg mL^{-1} PMS to examine the degradation property of ZIF-67-CT on rhodamine B (RhB) and methyl orange (MO) solutions described hereafter.

Supplementary Note 8: The feasibility of the facile scalable technology toward other MOFs and cellulose-based materials.

We performed the in situ growth of ZIF-8 and ZIF-67 on cotton textiles, linen, and filter paper to demonstrate the feasibility of the facile scalable technology toward other MOFs and cellulose-based materials. Supplementary Fig. 30 shows the photograph and SEM images of ZIF-8-CT. The color of ZIF-8-CT is white, which is attributed to the white color of ZIF-8 MOF coating on the fiber surface. The SEM images of the ZIF-8-CT at different magnifications show lots of nanosized ZIF-8 crystals are evenly distributed on the fibers, forming the ZIF-8 MOF coating. The XRD patterns show that the ZIF-8-CT has the same crystal characteristic peaks as the simulated crystal pattern of ZIF-8, confirming the ZIF-8 MOF coating was successfully formed on the fibers via in situ growth (Supplementary Fig. 31a). As shown in Supplementary Fig. 31b, the BET surface area of ZIF-8-CT is significantly increased to 94.9 m^2 g^{-1}, indicating that the high surface area and increased porosity are attributed to the in situ growth of ZIF-8 MOF coating on ZIF-8-CT.

In addition, we also fabricated the ZIF-67-Linen, ZIF-8-Linen, ZIF-67-Paper, and ZIF-8-Paper using the same method. The successful fabrication of the MOF-cellulose fibers was confirmed by SEM, XRD, and N_2 adsorption–desorption isotherms experiments (Supplementary Figs. 32, 33, 34, and 35), which have the same morphologies, crystal structures, and BET surface area with ZIF-67-CT and ZIF-8-CT. Besides, we carried out the in situ growth of UiO-66-NH_2 and MOF-303 on carboxymethylated cotton textiles, and the fabricated UiO-66-NH_2-CT and MOF-303-CT were measured using SEM and XRD (Supplementary Figs. 36 and 37). These results demonstrate this facile scalable method could well extend to other MOFs and cellulose fiber-based materials.

Supplementary Figures

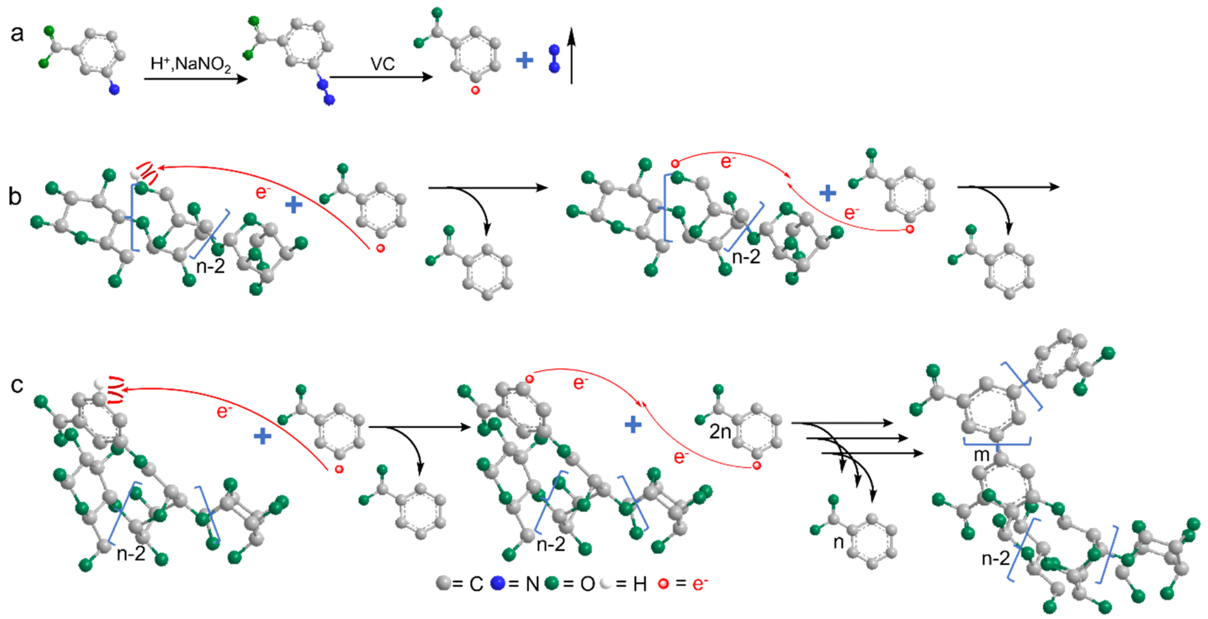

Supplementary Figure 1. Radical mechanism for the grafting of 3-aminobenzoic acid diazonium on the surface of cotton textiles via diazonium chemistry. a, The generation of aryl radical. b, The initiated homolytic reaction of hydroxyl groups by the generated aryl radicals. c, The formation of multilayer carboxyl polymer chain brushes via the covalent copolymerization of aryl radicals on the cellulose fiber surface.

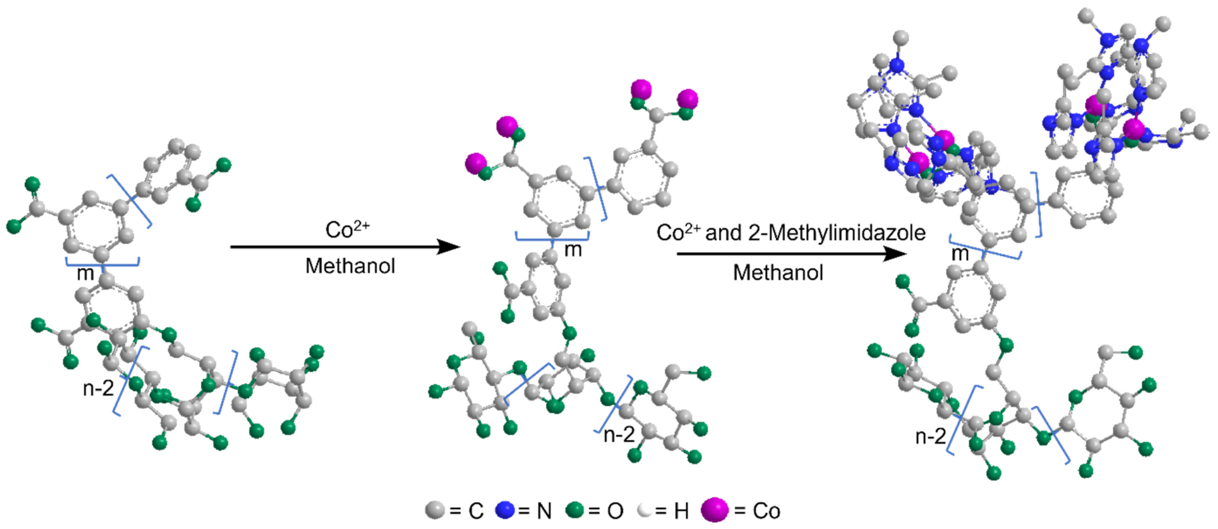

Supplementary Figure 2. Radical mechanism for the in situ growth of ZIF-67 on the carboxymethylated fiber surface of cotton textiles.

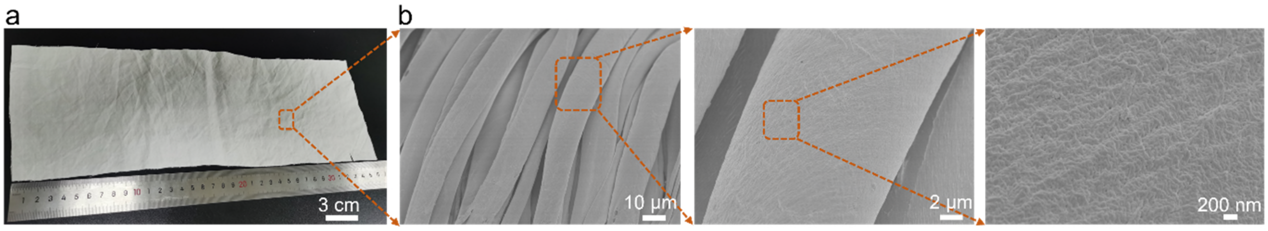

Supplementary Figure 3. The (a) photograph and (b) SEM images of pristine cotton fabric.

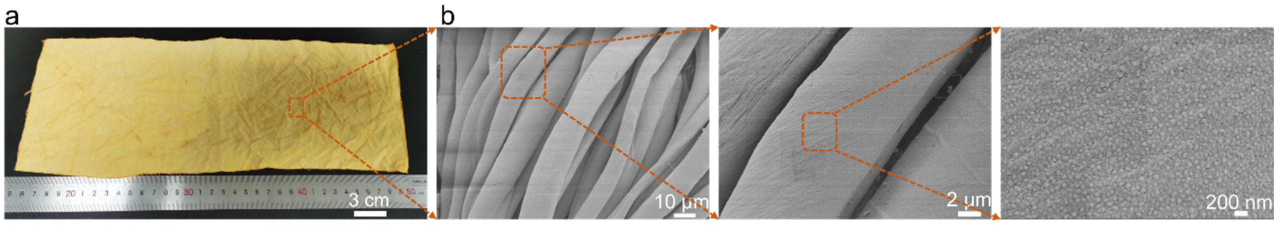

Supplementary Figure 4. The (a) photograph and (b) SEM images of carboxymethylated cotton fabric.

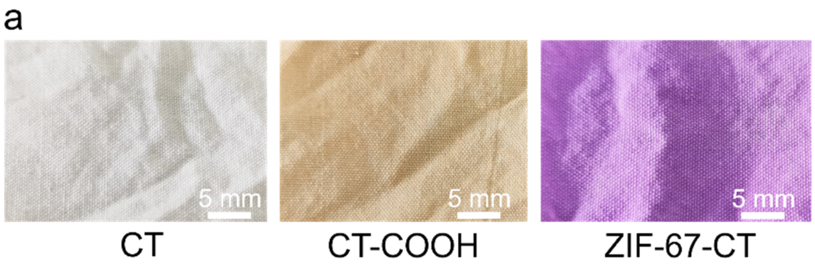

b

Samples	L^*	a^*	b^*	C^*	h^o
CF	93.34	-0.11	4.32	4.32	91.4
CF-COOH	76.16	12.32	35.75	37.81	70.98
ZIF-67-CF	53.3	16.2	-30.82	34.82	297.73

Supplementary Figure 5. The (a) photographs and (b) color values of CT, CT-COOH, and ZIF-67-CT.

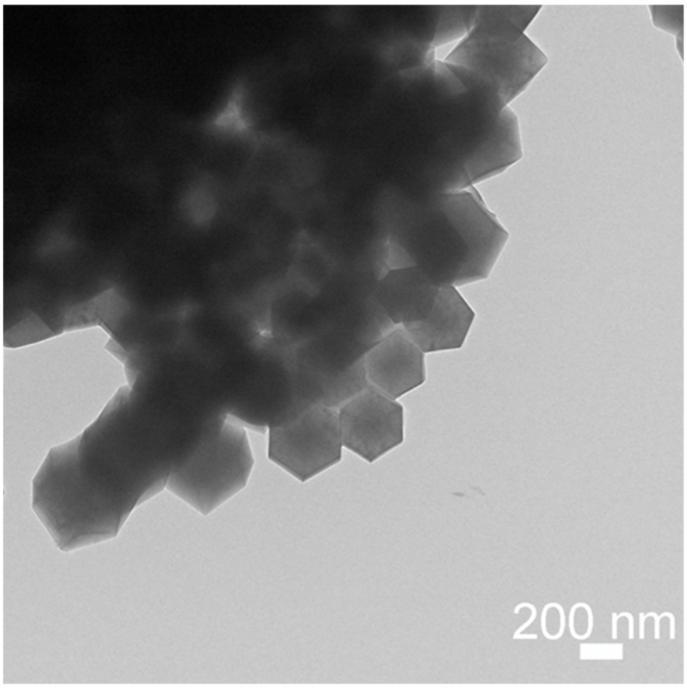

Supplementary Figure 6. The TEM image of ZIF-67 powders.

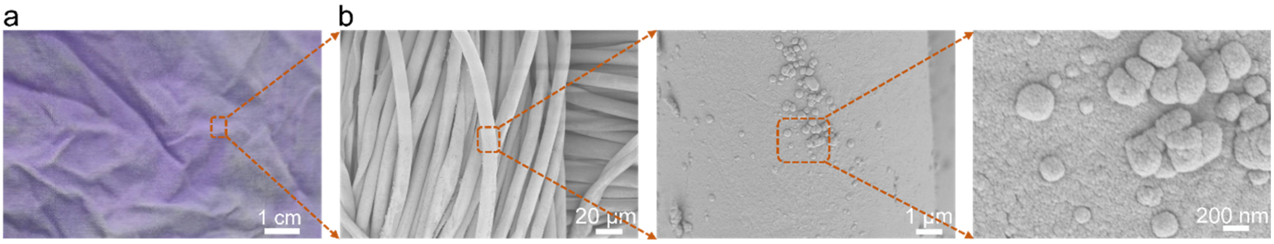

Supplementary Figure 7. The (a) photograph and (b) SEM images of ZIF-67 on pristine cotton fabric surface.

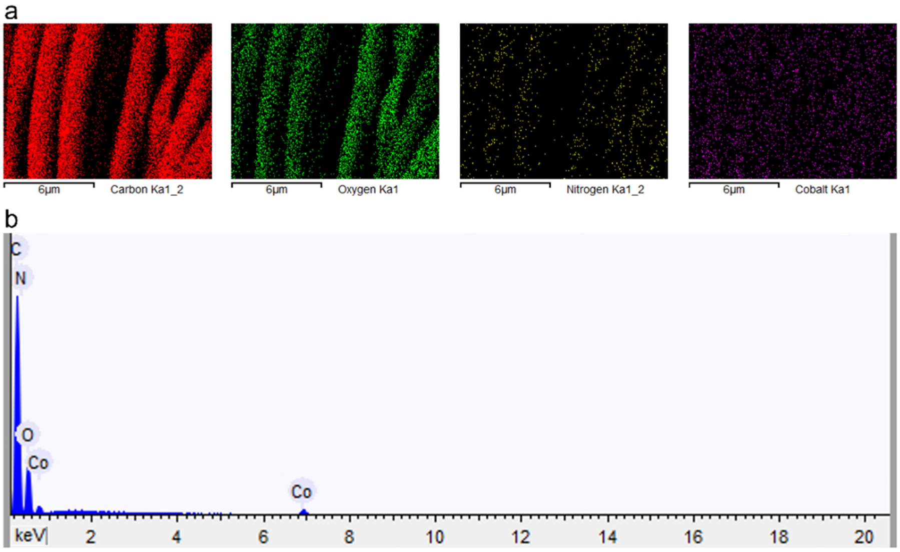

Supplementary Figure 8. The (a) EDS elemental mapping and (b) EDS spectrum of ZIF-67-CT.

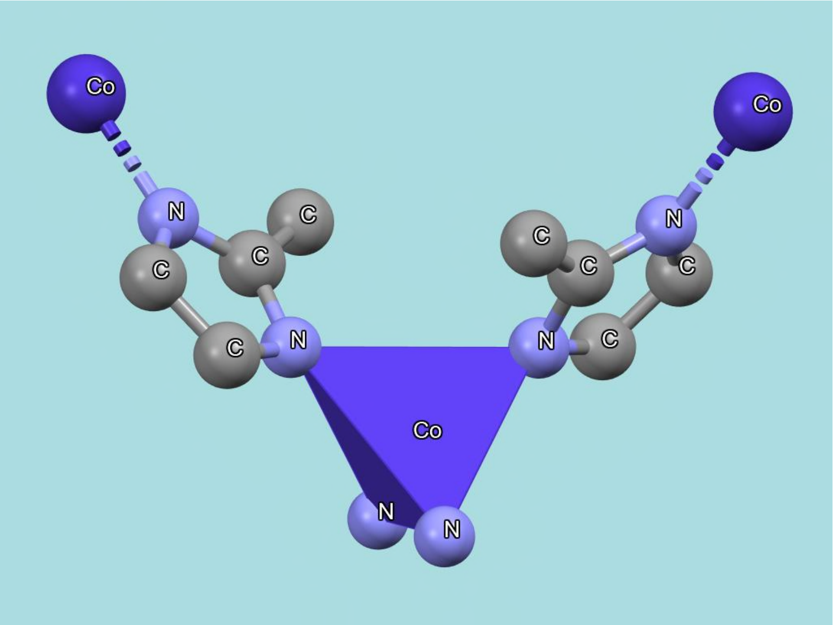

Supplementary Figure 9. The 3D simulated chemical structure of ZIF-67[25] (CCDC 671074: Experimental Crystal Structure Determination, 2008, DOI: 10.5517/ccqj9k2).

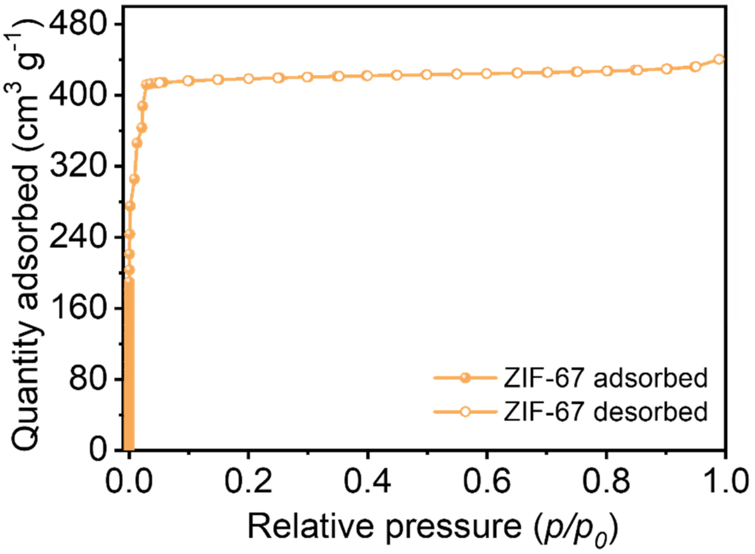

Supplementary Figure 10. The N_2 adsorption-desorption isotherm of ZIF-67 powder.

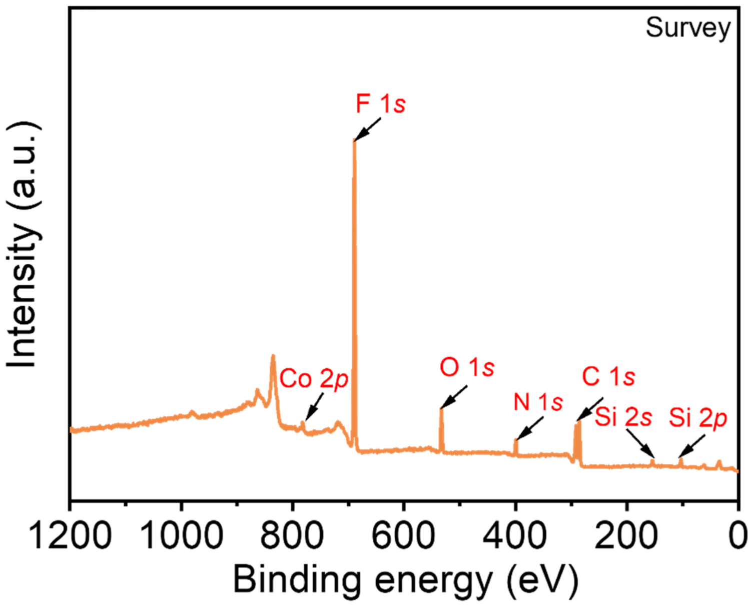

Supplementary Figure 11. The XPS spectrum of superhydrophobic ZIF-67-CT.

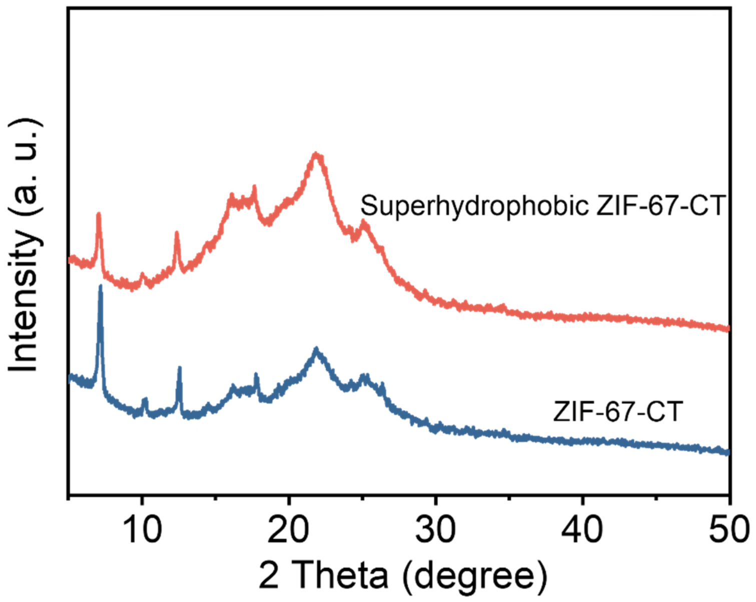

Supplementary Figure 12. The XRD patterns of ZIF-67-CT and superhydrophobic ZIF-67-CT.

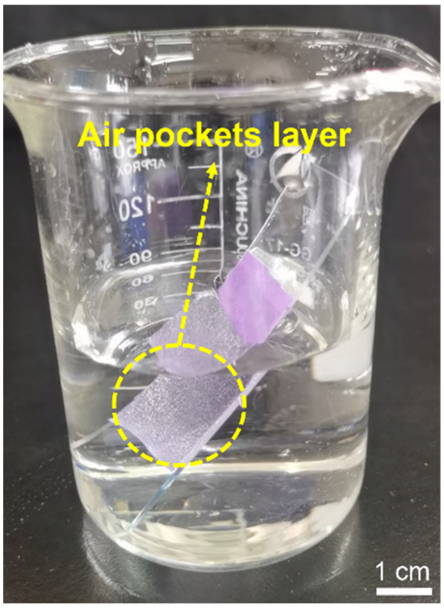

Supplementary Figure 13. Photograph of the superhydrophobic ZIF-67-CT in water.

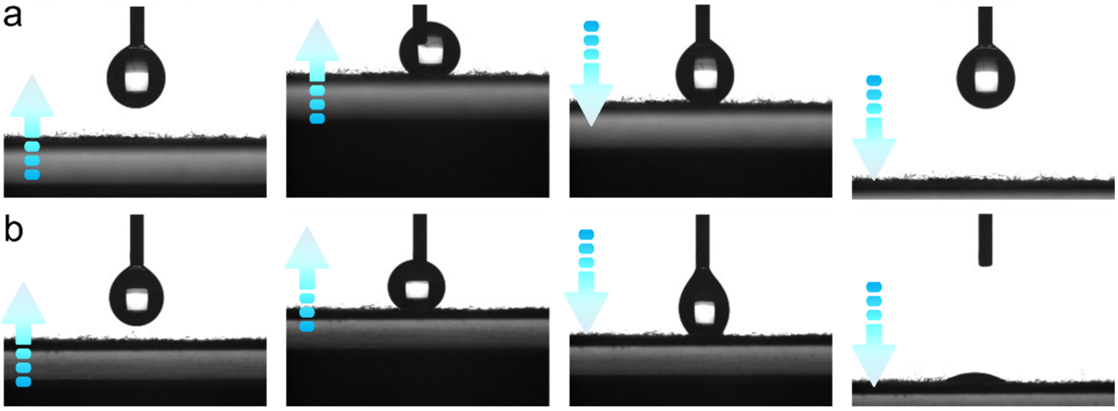

Supplementary Figure 14. The (a) water and (b) oil droplets adhesion behavior on superhydrophobic ZIF-67-CT surface.

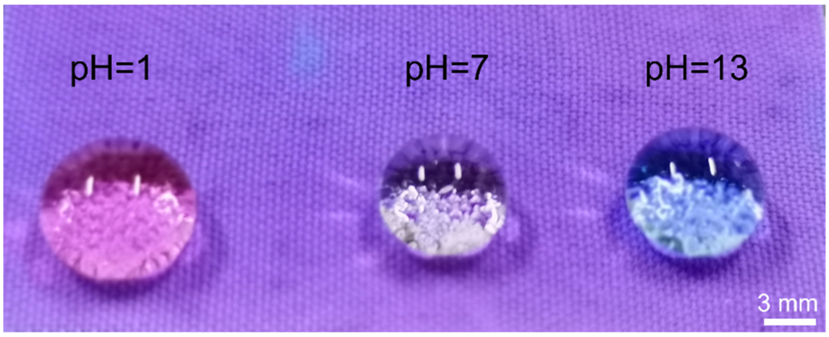

Supplementary Figure 15. Photograph of different pH values of water droplets on superhydrophobic ZIF-67-CT surface.

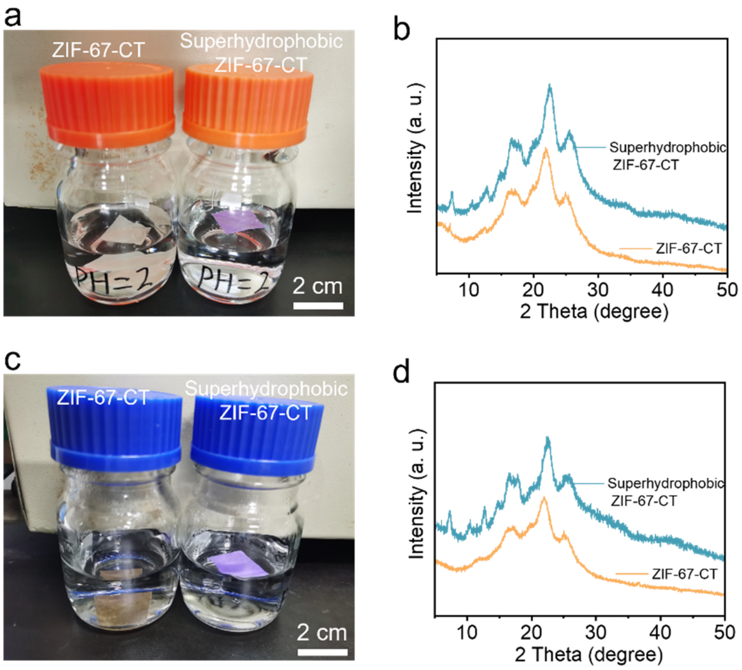

Supplementary Figure 16. Photographs of ZIF-67-CT and superhydrophobic ZIF-67-CT after exposure to a pH=2 (a) and pH=13 (c) solutions for 48 h at 30 °C. The corresponding XRD patterns of ZIF-67-CT and superhydrophobic ZIF-67-CT after exposure to pH=2 (b) and pH=13 (d) solutions for 48 h at 30 °C.

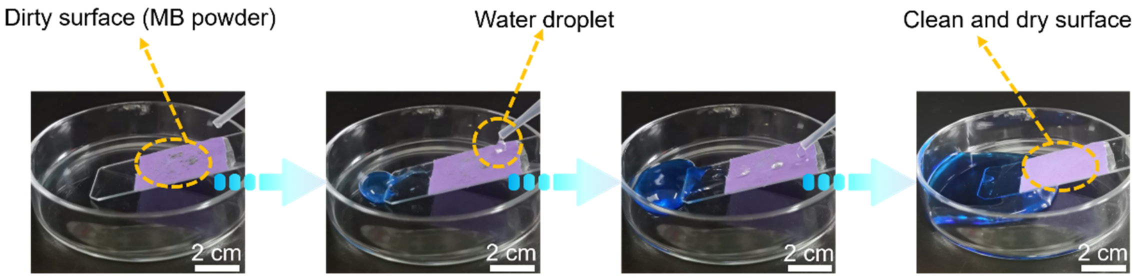

Supplementary Figure 17. The self-cleaning process of superhydrophobic ZIF-67-CT surface.

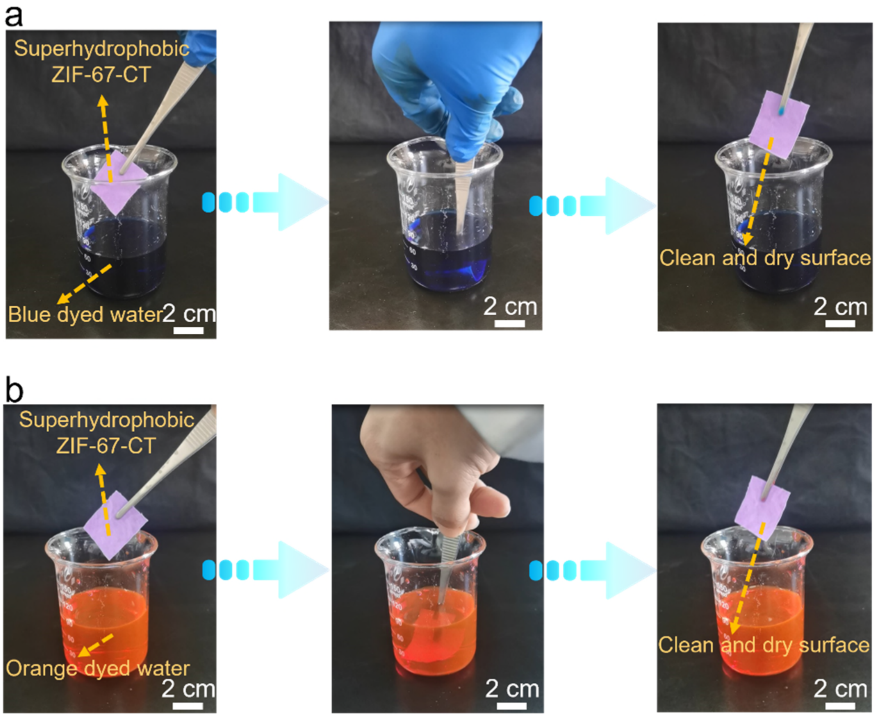

Supplementary Figure 18. The antifouling process of superhydrophobic ZIF-67-CT surface in (a) methylene blue solution and (b) methyl orange solutions.

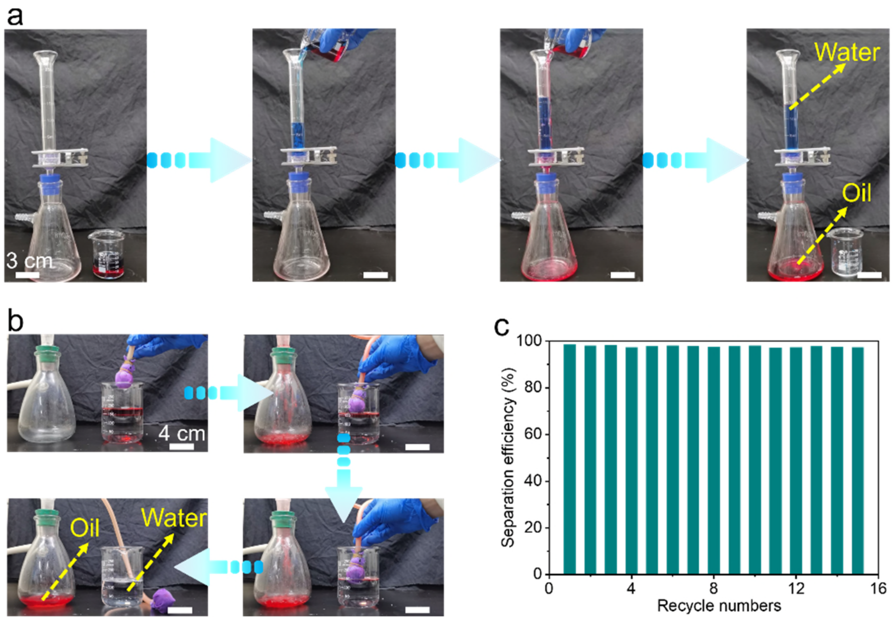

Supplementary Figure 19. Photographs of oil-water separation (a: carbon tetrachloride, b: petroleum ether) process of superhydrophobic ZIF-67-CT. (c) Recyclable separation ability of superhydrophobic ZIF-67-CT.

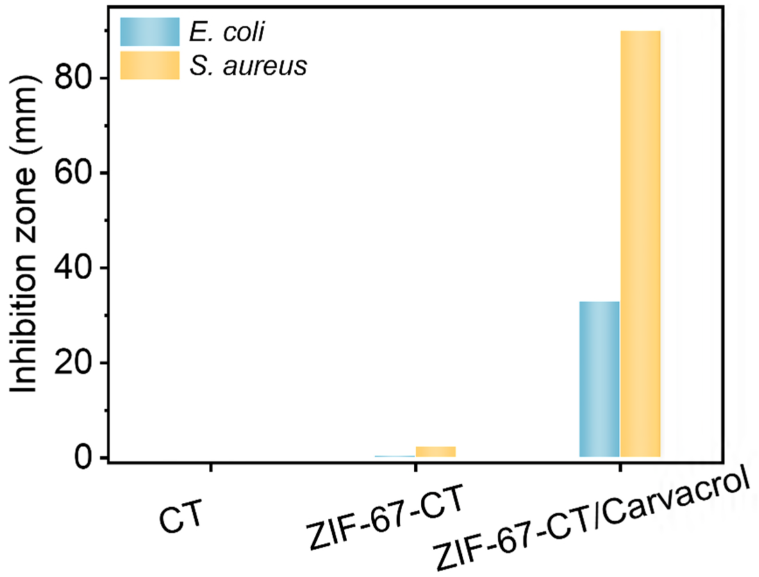

Supplementary Figure 20. Inhibition zones of CT, ZIF-67-CT, and ZIF-67-CT/Carvacrol against *E. coli* and *S. aureus*.

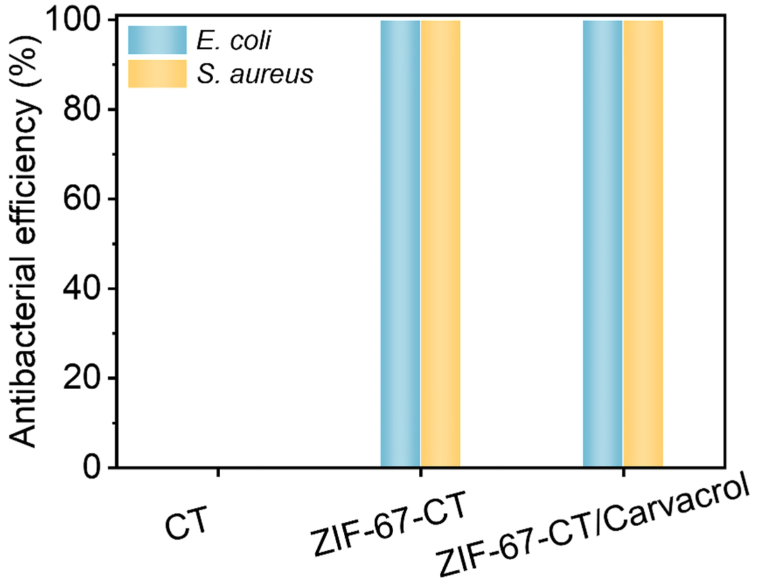

Supplementary Figure 21. The antibacterial efficiency of CT, ZIF-67-CT, and ZIF-67-CT/Carvacrol against *E. coli* and *S. aureus*.

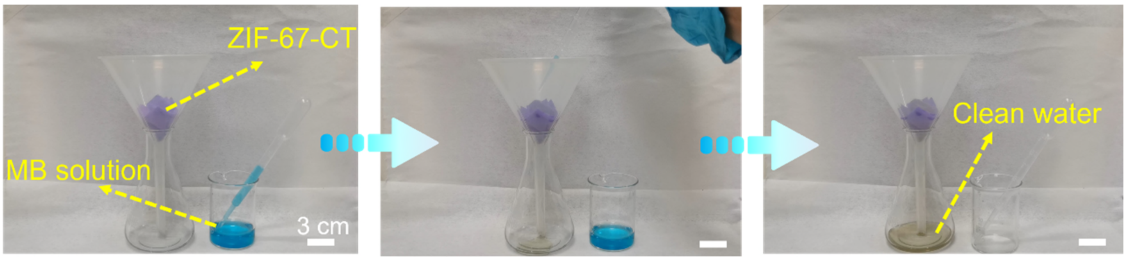

Supplementary Figure 22. The methylene blue solution degradation separation process of ZIF-67-CT.

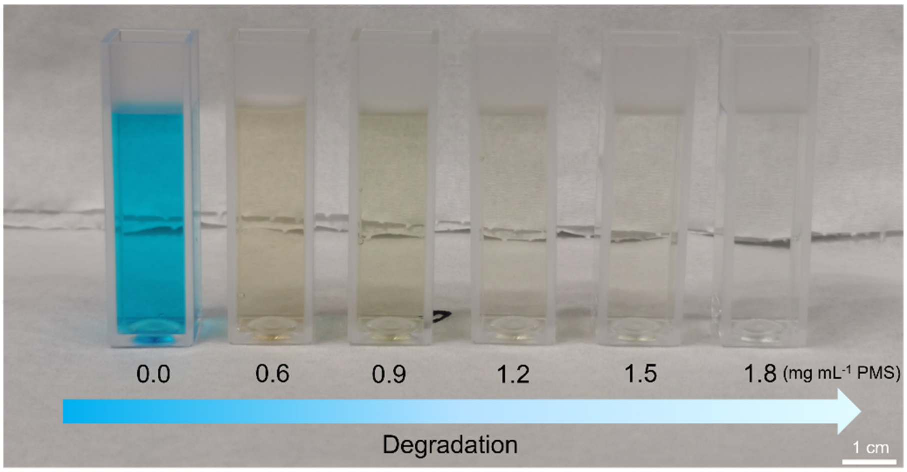

Supplementary Figure 23. The photograph of methylene blue solution with different concentrations of PMS after degradation by ZIF-67-CT.

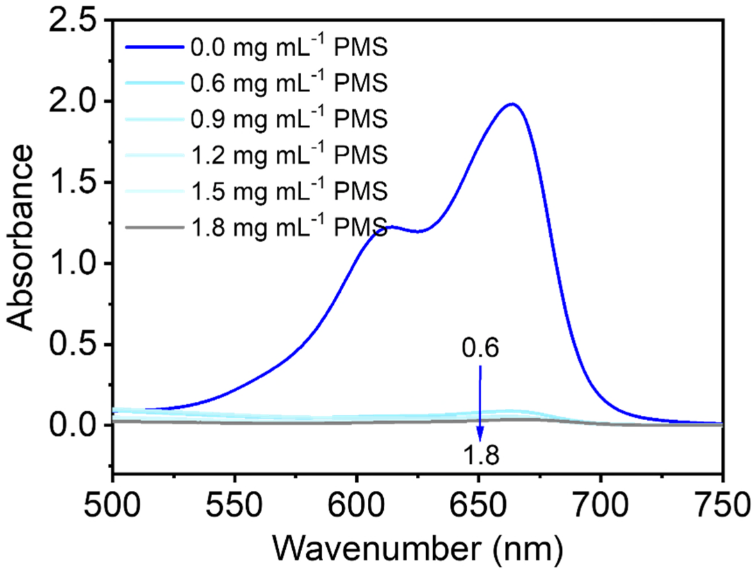

Supplementary Figure 24. The UV-vis absorption spectra of the methylene blue solution with different concentrations of PMS before and after degradation by ZIF-67-CT.

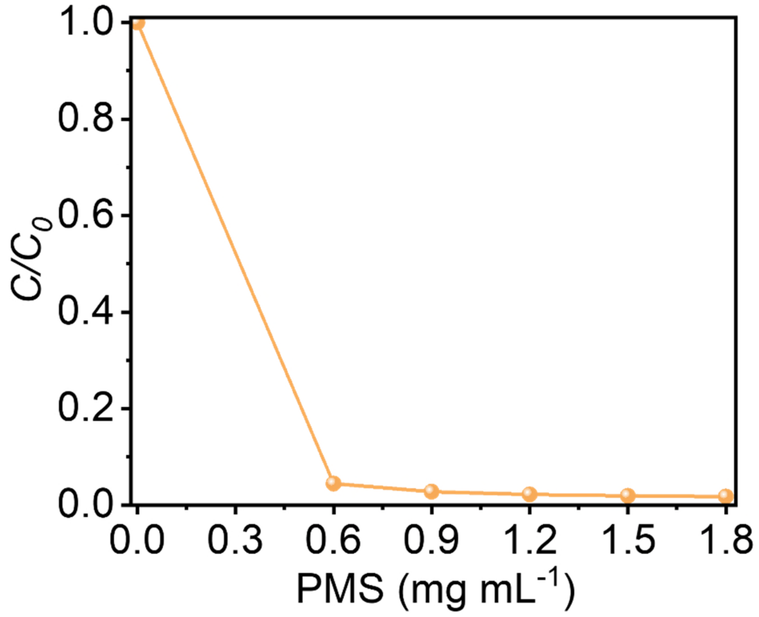

Supplementary Figure 25. The degradation performance of the methylene blue solution with different concentrations of PMS before and after degradation by ZIF-67-CT at room temperature (MB solution = 50 mg L^{-1}, PMS = 1.8 mg mL^{-1}, pH = 7).

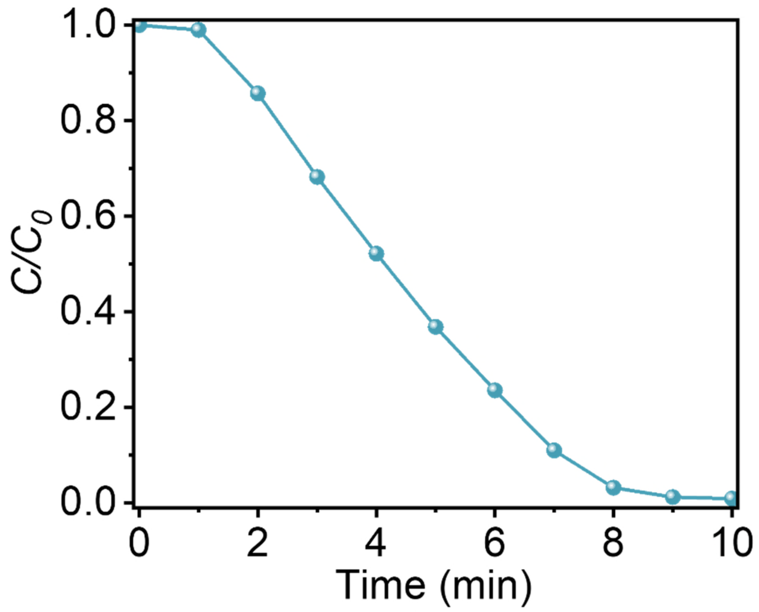

Supplementary Figure 26. The MB remove rate by ZIF-67-CT at room temperature (MB solution = 50 mg L^{-1}, PMS = 1.8 mg mL^{-1}, pH = 7).

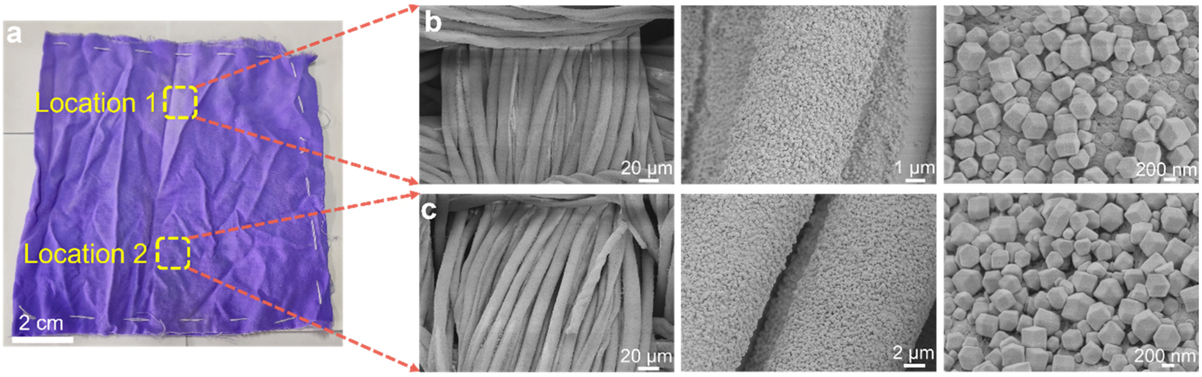

Supplementary Figure 27. The (a) photograph and (b and c) SEM images of ZIF-67-CT after laundering at varied locations.

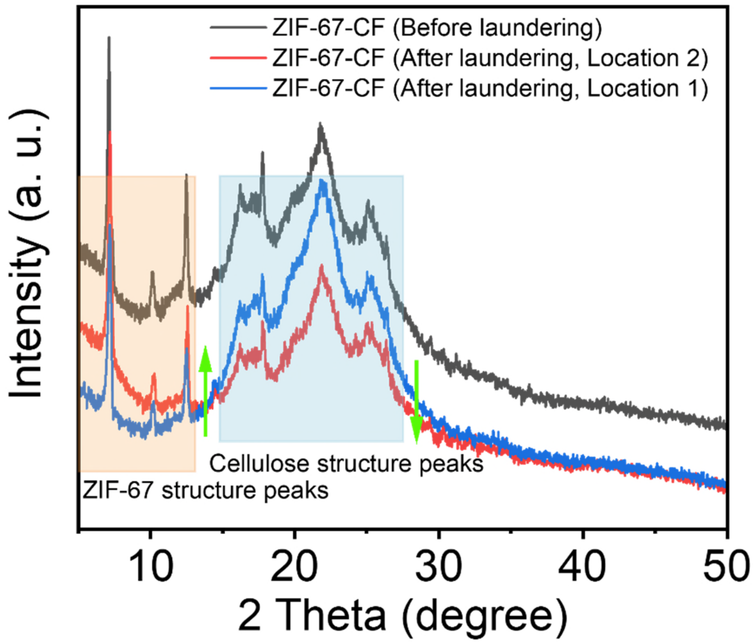

Supplementary Figure 28. The XRD patterns of ZIF-67-CT before and after laundering at varied locations. The upward green arrow indicates that the signal intensity of ZIF-67 structure peaks in location 2 is greater than that in location 1, and the downward green arrow indicates that the signal intensity of cellulose structure peaks in location 2 is smaller than that in location 1.

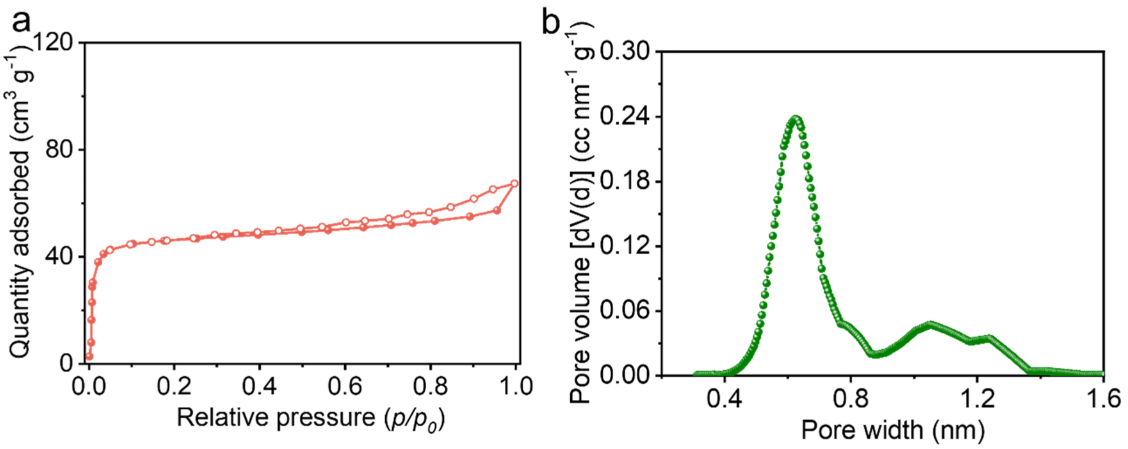

Supplementary Figure 29. The (a) N_2 adsorption–desorption isotherm and (b) pore width distribution of ZIF-67-CT after laundering.

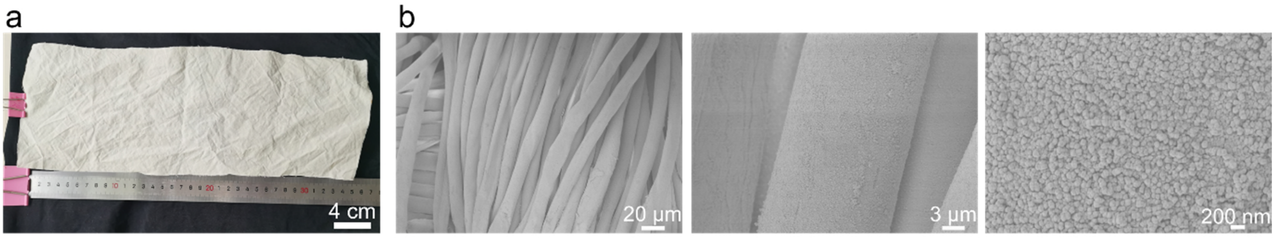

Supplementary Figure 30. The (a) photograph and (b) SEM images of ZIF-8-CT.

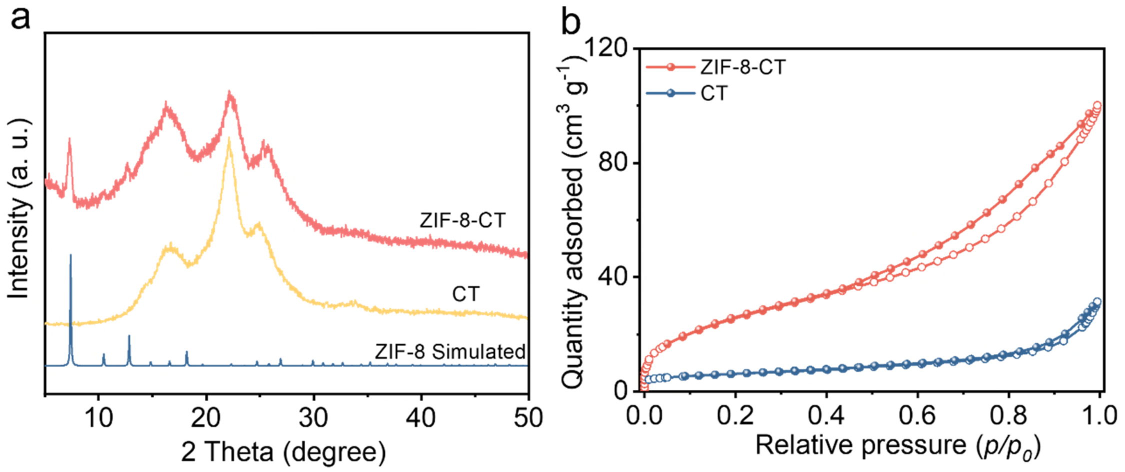

Supplementary Figure 31. The (a) XRD patterns and (b) N_2 adsorption–desorption isotherms of CT and ZIF-8-CT.

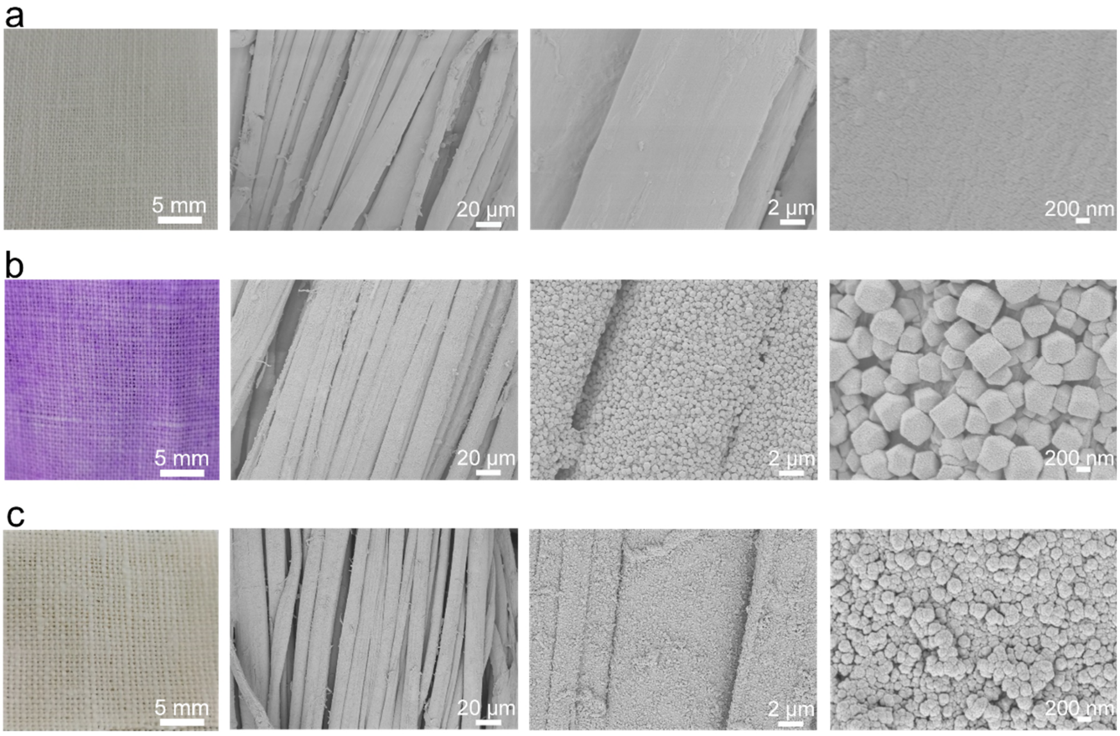

Supplementary Figure 32. The photographs and SEM images of (a) linen, (b) ZIF-67-Linen, and (c) ZIF-8-Linen.

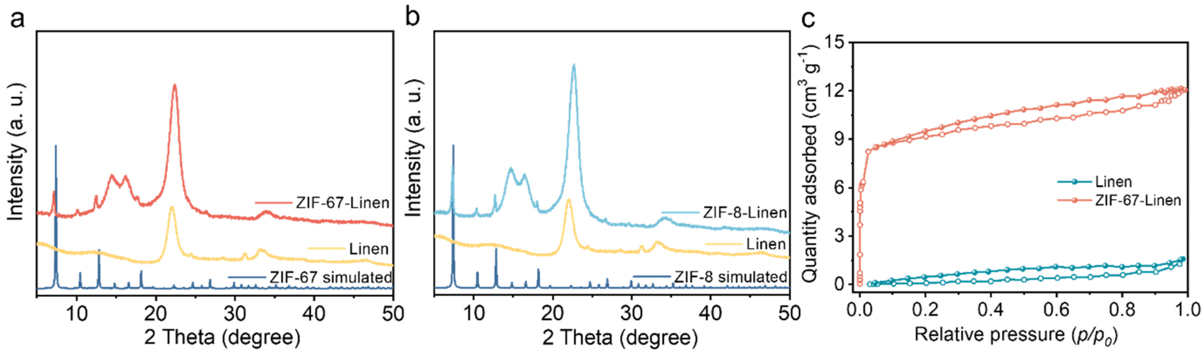

Supplementary Figure 33. The XRD patterns of linen, (a) ZIF-67-Linen, (b) ZIF-8-Linen, and (c) N_2 adsorption–desorption isotherms of linen and ZIF-67-Linen.

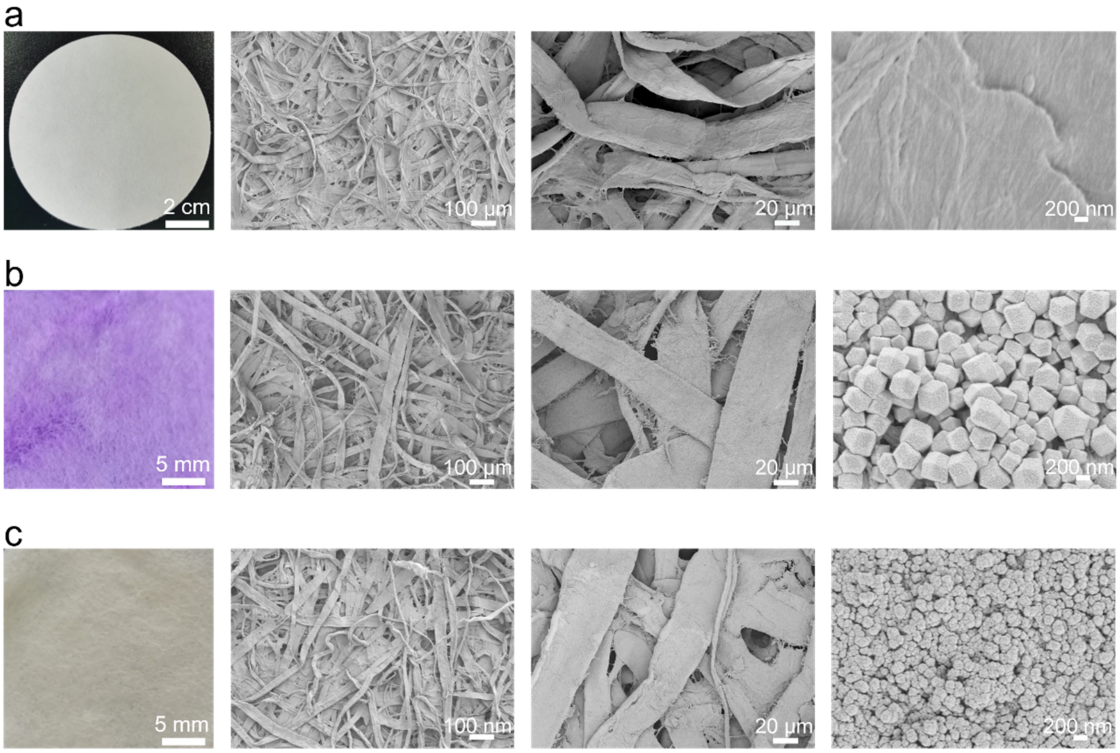

Supplementary Figure 34. The photographs and SEM images of (a) paper, (b) ZIF-67-Paper, and (c) ZIF-8-Paper.

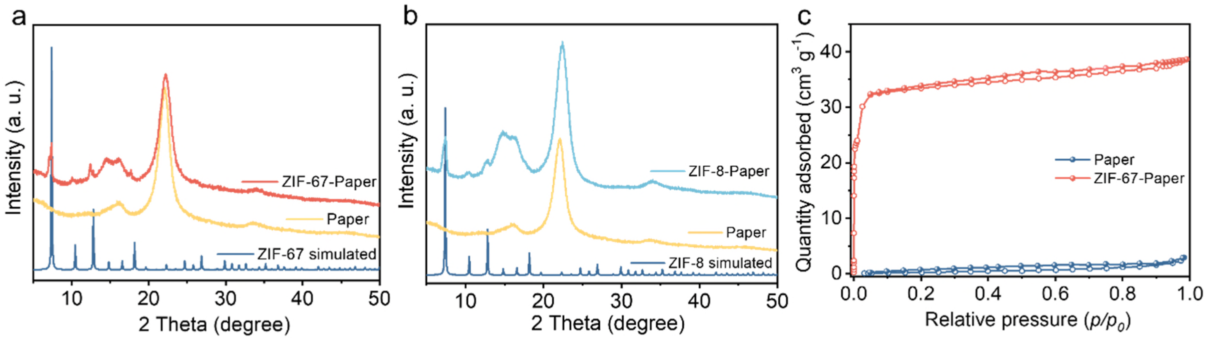

Supplementary Figure 35. The XRD patterns of paper, (a) ZIF-67-Paper and (b) ZIF-8-Paper, and (c) N_2 adsorption–desorption isotherms of paper and ZIF-67-Paper.

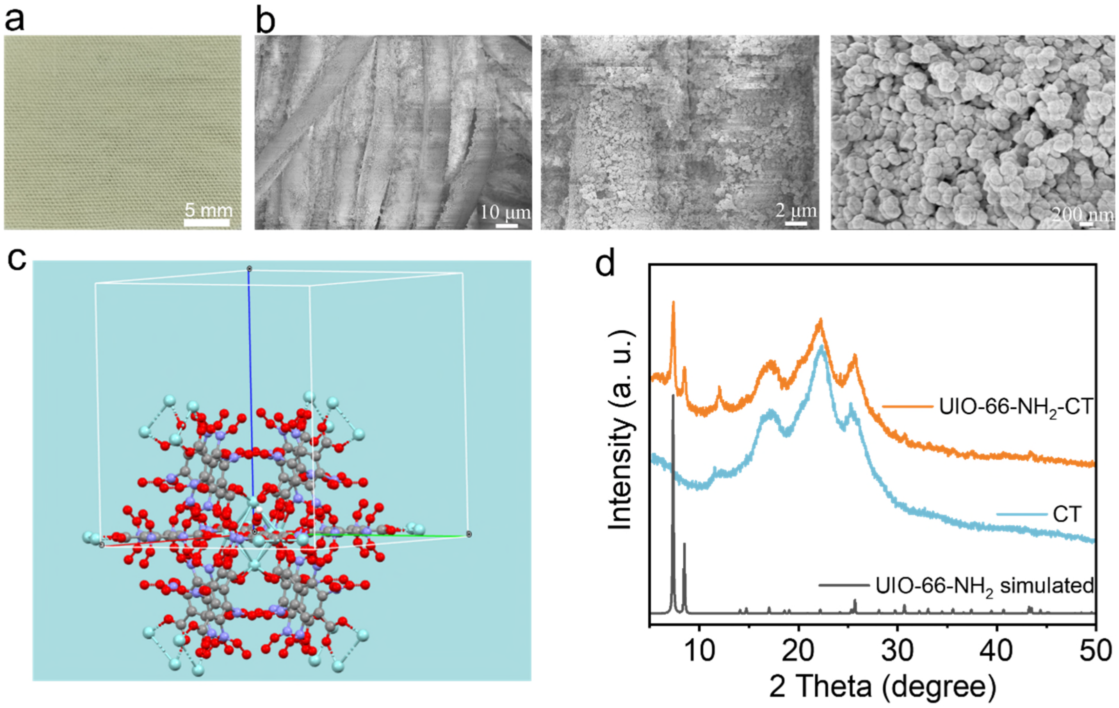

Supplementary Figure 36. The (a) photograph and (b) SEM images of UiO-66-NH_2-CT; (c) 3D simulated chemical structure of UiO-66-NH_2[26] (CCDC 1405751: Experimental Crystal Structure Determination, 2015, DOI: 10.5517/cc1j5st9); (d) XRD patterns of CT, UiO-66-NH_2-CT and simulated UiO-66-NH_2.

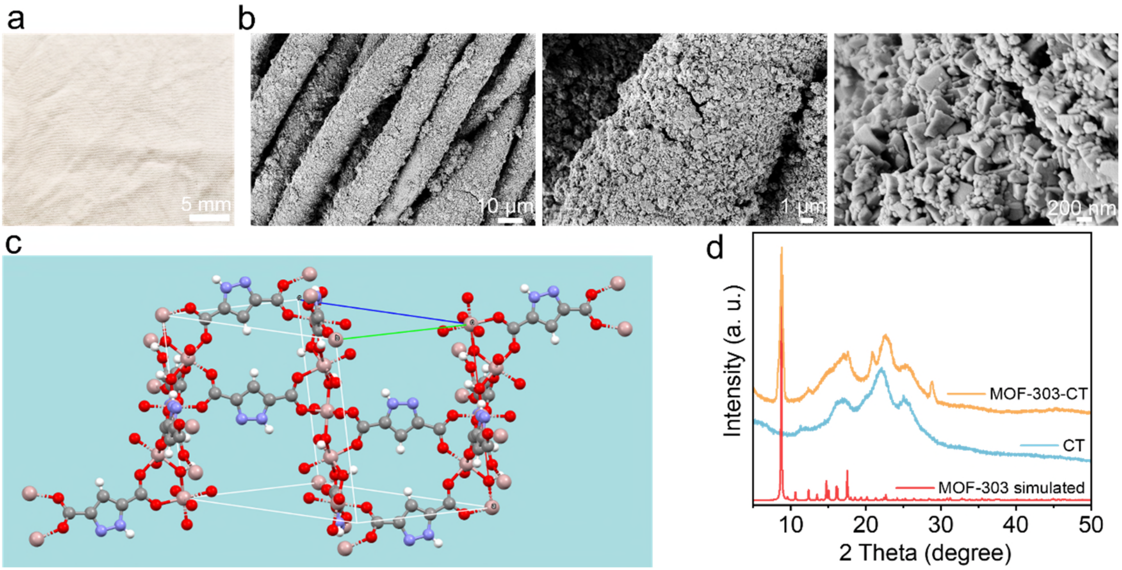

Supplementary Figure 37. The (a) photograph and (b) SEM images of MOF-303-CT; (c) 3D simulated chemical structure of MOF-303[27] (CCDC 2078717: Experimental Crystal Structure Determination, 2021, DOI: 10.5517/ccdc.csd.cc27s2dj); (d) XRD patterns of CT, MOF-303-CT and simulated MOF-303.

Supplementary References

1. Chen C. *et al.* Nonfluorinated multifunctional superhydrophobic cellulose sheet with polysaccharide B biopolymer-based hierarchical rough composite structure. *ACS Sustainable Chem. Eng.* **8**, 8505-8518 (2020).
2. Zeng T. *et al.* Facile fabrication of durable superhydrophobic and oleophobic surface on cellulose substrate via thiol-ene click modification. *Appl. Surf. Sci.* **493**, 1004-1012 (2019).
3. Gao S. *et al.* Co-solvent induced self-roughness superhydrophobic coatings with self-healing property for versatile oil-water separation. *Appl. Surf. Sci.* **459**, 512-519 (2018).
4. Cheng Q.-Y. *et al.* Robust and durable superhydrophobic cotton fabrics via a one-step solvothermal method for efficient oil/water separation. *Cellulose* **26**, 2861-2872 (2019).
5. Zhou H. *et al.* Durable superoleophobic–superhydrophilic fabrics with high anti-oil-fouling property. *RSC Adv.* **8**, 26939-26947 (2018).
6. Li W., Wang H. & Li Z. Preparation of golf ball-shaped microspheres with fluorinated polycaprolactone via single-solvent electrospraying for superhydrophobic coatings. *Prog. Org. Coat.* **131**, 276-284 (2019).
7. Zhang S. *et al.* Facile in situ synthesis of ZIF-67/cellulose hybrid membrane for activating peroxymonosulfate to degrade organic contaminants. *Cellulose* **28**, 3585-3598 (2021).
8. Qiao X. *et al.* Preparation of zeolitic imidazolate framework-67/wool fabric and its adsorption capacity for reactive dyes. *J. Environ. Manage.* **321**, 115972 (2022).
9. Cloutier M., Mantovani D. & Rosei F. Antibacterial coatings: challenges, perspectives, and opportunities. *Trends Biotechnol.* **33**, 637-652 (2015).
10. Choi A., Ben-Nissan B., Bendavid A. & Latella B. Mechanical behavior and properties of thin films for biomedical applications. *Thin Film Coatings for Biomaterials and Biomedical Applications*. Elsevier, 2016, pp 117-141.
11. Zhang B. *et al.* Biocompatible cyclodextrin-based metal–organic frameworks for long-term sustained release of fragrances. *Ind. Eng. Chem. Res.* **58**, 19767-19777 (2019).
12. Chen H. *et al.* Tuning the release rate of volatile molecules by pore surface engineering in metal-organic frameworks. *Chin. Chem. Lett.* **32**, 1988-1992 (2021).
13. Ahn Y.-Y. *et al.* Surface-loaded metal nanoparticles for peroxymonosulfate activation: Efficiency and mechanism reconnaissance. *Appl. Catal., B* **241**, 561-569 (2019).
14. Soltani T., Tayyebi A. & Lee B.-K. Quick and enhanced degradation of bisphenol A by activation of potassium peroxymonosulfate to $SO_4^{\cdot-}$ with Mn-doped $BiFeO_3$ nanoparticles as a heterogeneous Fenton-like catalyst. *Appl. Surf. Sci.* **441**, 853-861 (2018).
15. Mahouche-Chergui S., Gam-Derouich S., Mangeney C. & Chehimi M. M. Aryl diazonium salts: a new class of coupling agents for bonding polymers, biomacromolecules and

nanoparticles to surfaces. *Chem. Soc. Rev.* **40**, 4143-4166 (2011).

16. Vedrtnam A. & Sharma S. P. Study on the performance of different nano-species used for surface modification of carbon fiber for interface strengthening. *Composites, Part A* **125**, 105509 (2019).
17. Assresahegn B. D., Brousse T. & Bélanger D. Advances on the use of diazonium chemistry for functionalization of materials used in energy storage systems. *Carbon* **92**, 362-381 (2015).
18. Andrieux C. P. & Pinson J. The standard redox potential of the phenyl radical/anion couple. *J. Am. Chem. Soc.* **125**, 14801-14806 (2003).
19. Ryder C. R. *et al.* Covalent functionalization and passivation of exfoliated black phosphorus via aryl diazonium chemistry. *Nat. Chem.* **8**, 597-602 (2016).
20. Credou J., Volland H., Dano J. & Berthelot T. A one-step and biocompatible cellulose functionalization for covalent antibody immobilization on immunoassay membranes. *J. Mater. Chem. B* **1**, 3277-3286 (2013).
21. Anariba F., DuVall S. H. & McCreery R. L. Mono-and multilayer formation by diazonium reduction on carbon surfaces monitored with atomic force microscopy “scratching”. *Anal. Chem.* **75**, 3837-3844 (2003).
22. Adenier A. *et al.* Formation of polyphenylene films on metal electrodes by electrochemical reduction of benzenediazonium salts. *Chem. Mater.* **18**, 2021-2029 (2006).
23. Credou J., Faddoul R. & Berthelot T. One-step and eco-friendly modification of cellulose membranes by polymer grafting. *RSC Adv.* **4**, 60959-60969 (2014).
24. Zhang Y. *et al.* Fabrication of visual textile temperature indicators based on reversible thermochromic fibers. *Dyes Pigm.* **162**, 705-711 (2019).
25. Banerjee R. *et al.* High-throughput synthesis of zeolitic imidazolate frameworks and application to CO2 capture. *Science* **319**, 939-943 (2008).
26. Trickett C. A. *et al.* Definitive molecular level characterization of defects in UiO-66 crystals. *Angew. Chem. Int. Ed.* **54**, 11162-11167 (2015).
27. Hanikel N. *et al.* Evolution of water structures in metal-organic frameworks for improved atmospheric water harvesting. *Science* **374**, 454-459 (2021).
