# Supplementary material for: Scalable multifunctional MOFs-textiles via diazonium chemistry
Source: Nat Commun. 2024 Jun 21;15:5297. doi: 10.1038/s41467-024-49636-9 (PMC11192900; doi:10.1038/s41467-024-49636-9)
Supplement: Supplementary file 3 — Description of Additional Supplementary Files [file 41467_2024_49636_MOESM3_ESM.pdf]

Description of Additional Supplementary Files

File Name: Supplementary Movie 1
Description: The oil-water separation process of superhydrophobic ZIF-67-CT.

File Name: Supplementary Movie 2
Description: Laundering process of ZIF-67-CT based on the international standard ISO 105 C10.

File Name: Supplementary Movie 3
Description: Displaying process of the large-scale ZIF-67-CT (30 m × 0.25 m).
